# A Thought-Provoking Case of Successfully Treated Carcinoma of the Head of the Pancreas with Metachronous Lung Metastasis: Impact of Distal Spleno-Renal Shunt for Regional Invasion on Long-Term Period after Pancreaticoduodenectomy

**DOI:** 10.1155/2021/6689419

**Published:** 2021-05-28

**Authors:** Ryuhei Aoyama, Tomohide Hori, Hidekazu Yamamoto, Hideki Harada, Michihiro Yamamoto, Masahiro Yamada, Takefumi Yazawa, Ben Sasaki, Masaki Tani, Asahi Sato, Hikotaro Katsura, Yasuyuki Kamada, Ryotaro Tani, Yudai Sasaki, Masazumi Zaima

**Affiliations:** Department of Surgery, Shiga General Hospital, Moriyama, Japan

## Abstract

When performing pancreaticoduodenectomy with resection of the confluence of the superior mesenteric vein and portal vein, division of the splenic vein may cause sinistral portal hypertension resulting in gastrointestinal bleeding, splenic congestion, and hypersplenism. To prevent these adverse events, it is important to intentionally decompress the splenic vein. This report is of a 68-year-old woman with stage IA carcinoma of the head of the pancreas who survived for more than six years following tumor resection and pancreaticoduodenectomy and distal splenorenal shunt. A 68-year-old woman was diagnosed with carcinoma of the head of the pancreas that involved the confluence of the superior mesenteric vein, portal vein, and splenic vein. No unresectable cancer sites or distant metastases were detected. Pancreaticoduodenectomy with resection of the confluence of the superior mesenteric vein and portal vein was performed. The superior mesenteric vein and portal vein were anastomosed in the end-to-end fashion, and the remnant splenic vein was anastomosed to the superior aspect of the left renal vein in the end-to-side fashion. At 22 months after the initial surgery, the patient underwent partial lung resection for a metachronous lung metastasis. For 6 years after the initial surgery, the venous reconstructions have maintained their patency without any obstruction of splenic venous flow, and the patient has remained in good health without further metastases or recurrences. This case has shown the importance of early diagnosis of carcinoma of the head of the pancreas, as appropriate and timely surgical management can result in good outcome. This patient responded well and remains alive six years following pancreaticoduodenectomy and preservation of the spleen with the use of a distal splenorenal shunt.

## 1. Introduction

Pancreas cancer is the fourth leading cause of cancer-related death in developed countries [1], and the frequency currently increases with unknow reason [2]. Pancreatic cancer often accompanies with distant metastasis and generally shows poor prognosis [3–6]. Pancreatic cancer remains one of the most lethal malignancies with a 5-year survival rate of 5–10% [7, 8]. Pancreatic cancer is often already incurable at the time of diagnosis and have a median overall survival of 6–12 months even with palliative chemotherapy [7]. The risk of postoperative recurrence is high, and a 5-year survival rate after surgery was documented as approximately 30% [7]. Hence, multidisciplinary treatment should be considered [8].

Pancreatic cancer located in the pancreatic head and/or uncus easily invades the superior mesenteric vein and/or portal vein [9–11]. Although venous reconstruction of the superior mesenteric vein and portal vein is widely accepted, it remains controversial whether there is a validity of intentional decompression of the splenic vein in cases requiring diversion of the splenic vein [12–20].

W. Dean Warren (1924-1989) was a pioneer in surgery for portal hypertension [21], and he focused on therapeutic potential of distal splenorenal shunt for portal hypertension and variceal bleeding [22–26]. He first described surgical procedure using distal splenorenal shunt in 1967 [27], and “the Warren shunt,” is a vascular surgery of distal splenorenal shunt. Briefly, the splenic vein is detached from the superior mesenteric vein and portal vein and is subsequently reattached to the left renal vein [21].

Division and ligation of the splenic vein during pancreaticoduodenectomy is often associated with the development of sinistral portal hypertension, which results in gastrointestinal bleeding, splenic congestion, and hypersplenism over the long term [12, 13]. Therefore, optimal management of the splenic vein is required [13]. The individual vascular anatomy dictates whether intentional preservation of the splenic venous drainage via the inferior mesenteric vein or other venous tributaries is sufficient to prevent sinistral portal hypertension [13, 14]; some surgeons suggest that splenic venous reconstruction is important for long-term splenic decompression [15–20].

This report describes a 68-year-old woman with stage IA carcinoma of the head of the pancreas who survived for more than six years following tumor resection and pancreaticoduodenectomy with the use of a distal splenorenal shunt to preserve the spleen.

## 2. Case Presentation

**Figure 1:**
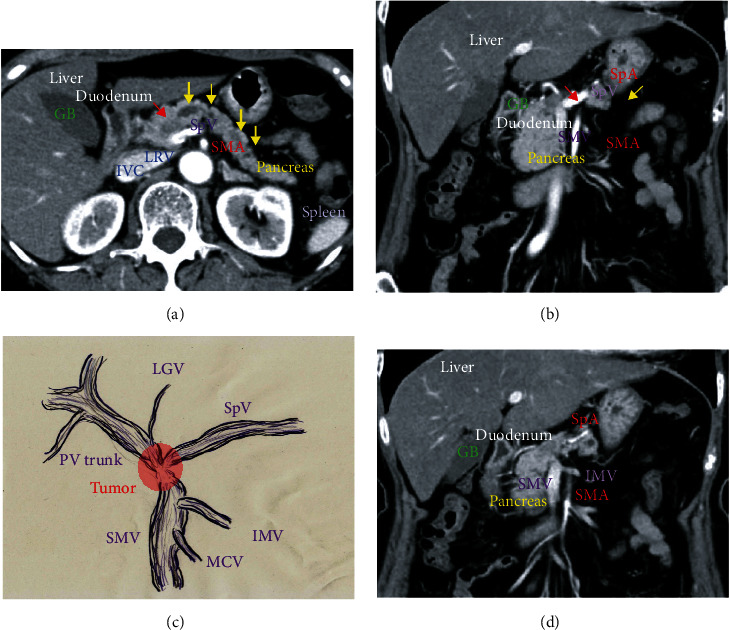
Pancreatic cancer with regional invasion into vessels. (a, b) Enhanced computed tomography shows a hypovascular mass in the pancreatic head (red arrow) and marked dilation of the main pancreatic duct of the distal pancreatic parenchyma (yellow arrows). (c) The pancreatic tumor (red area) markedly involves the confluence of the superior mesenteric vein, portal venous trunk, and splenic vein. (d) The inferior mesenteric vein flows into the superior mesenteric vein, not the splenic vein. There is no evidence of cancer at unresectable sites or distant metastases. GB: gallbladder; IMV: inferior mesenteric vein; LGV: left gastric vein; LRV: left renal vein; IVC: inferior vena cava; MCV: middle colic vein; SMA: superior mesenteric artery; SMV: superior mesenteric vein; SpA: splenic artery; SpV: splenic vein.

A 68-year-old woman was referred to our hospital for the investigation of elevated serum levels of amylase and lipase. Enhanced computed tomography (CT) detected a hypovascular mass (18 mm diameter) in the pancreatic head (Figures 1(a) and 1(b)). The main pancreatic duct of the distal pancreatic parenchyma was substantially dilated (Figures 1(a) and 1(b)). Endoscopic retrograde and magnetic resonance cholangiopancreatographies showed severe stenosis of the main pancreatic duct due to the pancreatic tumor, but biliary obstruction was not observed. The diagnosis of carcinoma of the head of the pancreas was made based on the histopathologic examination of an endoscopic ultrasound-guided fine-needle aspiration biopsy. Dynamic CT clearly demonstrated that the carcinoma of the head of the pancreas had invaded the superior mesenteric vein, portal venous trunk, and splenic vein (Figure 1(c)). The inferior mesenteric vein flowed into the superior mesenteric vein rather than the splenic vein (Figure 1(d)), suggesting that the inferior mesenteric vein was not a viable splenic venous drainage route. There were no findings indicating cancer at an unresectable site or distant metastases. Neoadjuvant chemotherapy was not employed, and a subtotal stomach-preserving pancreaticoduodenectomy with lymphadenectomy was performed. Histopathological assessments revealed that a moderately differentiated invasive ductal carcinoma had invaded the venous wall of the confluence of the superior mesenteric vein, portal venous trunk, and splenic vein. Pancreatic cancer measuring 1 to 2 cm is categorized as T1c, and the cancer categorized as T1cN0M0 stage IA in accordance with the tumor-node-metastasis classification [28], although pancreatic cancer growing outside the pancreas and into nearby major blood vessels is categorized as T4 and stage III according to staging system of American Cancer Society [29].

**Figure 2:**
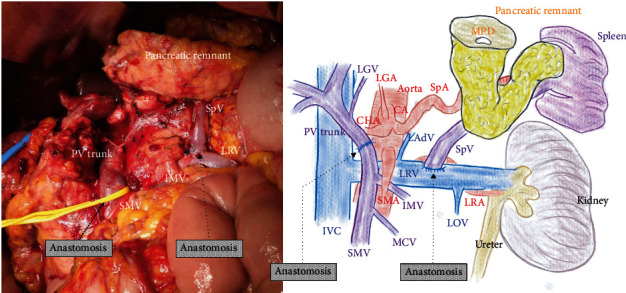
The distal splenorenal shunt. The confluence of the superior mesenteric vein, portal vein, and splenic vein are resected *en bloc*. The superior mesenteric vein and portal venous trunk are anastomosed in the end-to-end fashion. The remnant splenic vein is anastomosed to the superior aspect of the left renal vein in the end-to-side fashion. CA: celiac artery; CHA: common hepatic artery; IMV: inferior mesenteric vein; LAdV: left adrenal vein; LGA: left gastric artery; LGV: left gastric vein; LOV: left ovarian vein; LRA: left renal artery; LRV: left renal vein; IVC: inferior vena cava; MCV: middle colic vein; MPD: main pancreatic duct; PV: portal vein; SMA: superior mesenteric artery; SMV: superior mesenteric vein; SpA: splenic artery; SpV: splenic vein.

**Figure 3:**
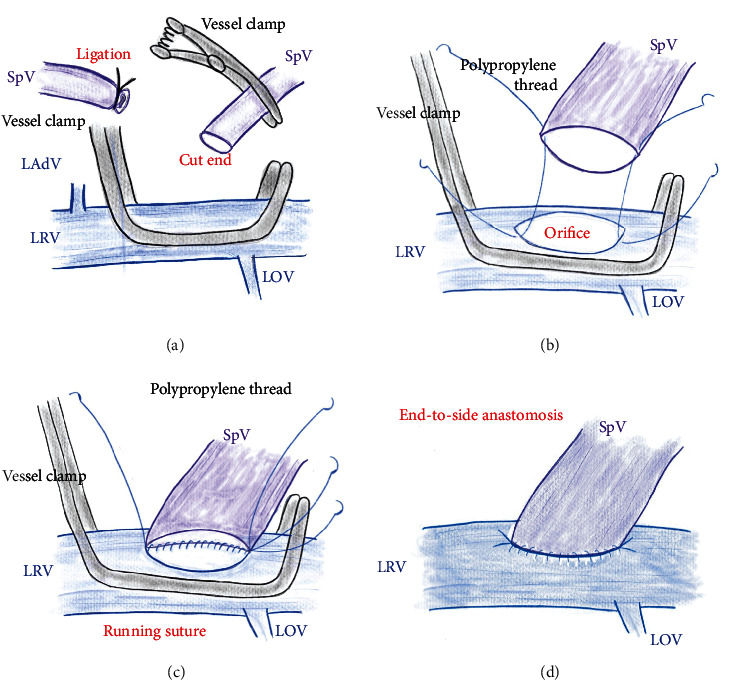
Surgical procedures. (a) The splenic vein is ligated and cut, and the splenic vein is temporarily clamped. The left renal vein is partially clamped. (b) A venous orifice is made on the superior aspect of the LRV. The splenic vein and left renal vein are set in the end-to-side fashion by bilateral fixation sutures. (c, d) The splenic vein is anastomosed to the left renal vein in the end-to-side fashion by unabsorbable polypropylene. LAdV: left adrenal vein; LOV: left ovarian vein; LRV: left renal vein; SpV: splenic vein.

As imaging examinations revealed that the tumor involved the confluence of the superior mesenteric vein, portal venous trunk, and splenic vein (Figures 1(c) and 1(d)), *en bloc* resection of the confluence of these veins was performed, with simultaneous venous reconstructions (Figure 2). The lower margin of the distal pancreas was incised, and the pancreas was rotated cranially to expose the posterior surface of the splenic vein. The splenic vein was then ligated at the confluence of the superior mesenteric vein and portal vein and dissected toward the pancreatic tail to obtain a sufficient length for anastomosis. The splenic vein was divided as near as possible to the junction of the superior mesenteric vein and portal vein and temporarily clamped (Figure 3(a)). The left renal vein was then prepared for anastomosis on the left side of the superior mesenteric artery and partially clamped (Figure 3(a)). An anastomosis orifice was created on the superior aspect of the left renal vein rather than the ventral aspect (Figure 3(b)). After widening the cut end of the splenic vein by making a small slit in its heel side, the splenic vein and left renal vein were anastomosed in the end-to-side fashion by bilateral fixation sutures (6-0 Prolene; Ethicon, Inc., Cincinnati, USA) (Figure 3(b)). Subsequently, the remnant splenic vein was anastomosed to the superior aspect of the left renal vein in the end-to-side fashion by unabsorbable polypropylene (6-0 Prolene) (Figures 3(c) and 3(d)). After the specimen was removed, the superior mesenteric vein and portal venous trunk were anastomosed in the usual end-to-end fashion by running sutures using unabsorbable polypropylene (5-0 Prolene). The operative time was 348 minutes. The intraoperative blood loss volume was 1,294 mL, and no blood transfusion was required. Although there was temporary ulceration of the gastrojejunostomy, categorized as grade II in accordance with the Clavien-Dindo classification [30], the postoperative course was uneventful.

Histopathological assessments of resected specimen revealed that moderately differentiated invasive ductal carcinoma clearly invaded into venous wall of the confluence of the superior mesenteric vein, portal venous trunk, and splenic vein. Adjuvant chemotherapy with S-1 was administered for 6 months postoperatively. At 22 months after the initial surgery, ground-glass opacity (8 mm in size) was graphically detected in the right lung. The patient underwent partial lung resection for a right lung tumor that was pathologically diagnosed as a lung metastasis originated from the pancreatic cancer (i.e., metachronous metastasis), although graphical and surgical curability were obtained. Chemotherapy with S-1 was resumed for 1 year after the lung resection. The patient has been in good health, with no other oncologic metastases detected during the 6 years since the initial surgery.

**Figure 4:**
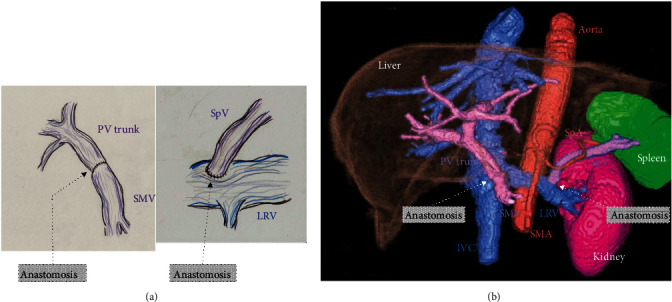
Long-term patency of the distal splenorenal shunt. (a) Schemas for venous reconstructions during pancreaticoduodenectomy. (b) Three-dimensional imagery at 5 years and 6 months after the initial surgery shows that the distal splenorenal shunt has maintained good patency. The estimated splenic volume is 35 mL. LRV: left renal vein; IVC: inferior vena cava; PV: portal vein; SMA: superior mesenteric artery; SMV: superior mesenteric vein; SpA: splenic artery; SpV: splenic vein.

Dynamic CT and upper esophagogastric endoscopy performed 5 years and 6 months after the initial surgery revealed that all venous reconstructions had maintained good patency (Figure 4), and there were no esophageal varices or congestive gastropathy. The platelet counts before surgery and at 5 years and 6 months after the initial surgery were 221,000/*μ*L and 235,000/*μ*L, respectively. The estimated splenic volumes before surgery and at 5 years and 6 months after the initial surgery were 38 mL and 35 mL, respectively.

## 3. Discussion

Intentional decompression of the splenic vein after division of this vein is still controversial [12–20], and a creation of distal splenorenal shunt requires vascular surgical skill. Moreover, postoperative long-term effect of distal splenorenal shunt is unknown, because pancreatic cancer generally accompanies with distant metastasis and shows very poor prognosis [3–6]. Our thought-provoking case suggested intentional decompression of the splenic vein should be considered for the long-term postoperative course, and distal splenorenal shunt during pancreaticoduodenectomy is a powerful tool for carcinoma of the head of the pancreas with regional invasion.

Although venous reconstruction of the superior mesenteric vein and portal vein during pancreaticoduodenectomy for pancreatic cancer is widely accepted, intentional decompression of the splenic vein in case when division of the splenic vein is needed is still controversial [12–20]. It has been clearly demonstrated that division of the splenic vein during pancreaticoduodenectomy causes sinistral portal hypertension, which results in intractable symptoms (e.g., gastrointestinal bleeding, splenic congestion, and hypersplenism) in the long term [12, 13]. Hence, many surgeons focused on sinistral portal hypertension after pancreaticoduodenectomy with resection of the confluence of superior mesenteric and portal veins, because only a little was previously known [18]. It is currently clarified that pancreaticoduodenectomy with splenic venous division causes variceal formation, bleeding, and thrombocytopenia [18], and therefore, the splenic venous flow is intentionally reconstructed if pancreaticoduodenectomy accompanies with the splenic venous diversion [15–20].

As pancreatic cancer generally has a very poor prognosis [3–6], many surgeons have not payed much attention to the consequence of sinistral portal hypertension on the long-term postoperative course. However, multimodality therapy has improved the prognosis of pancreatic cancer [31–33], and intentional decompression of the splenic vein is currently considered important for the long-term postoperative course.

A native vein may be the optimal candidate as a drainage route of the splenic vein. The inferior mesenteric vein is useful as a drainage route from the splenic vein [13, 34, 35], but the inferior mesenteric vein only works as a drainage route when it is connected to the splenic vein. In contrast to other opinions, others suggest that intentional preservation of the inferior mesenteric vein might not prevent splenic congestion [14], as the left gastric vein, middle colic vein, and superior right colic vein arcade may provide adequate drainage from the splenic vein and prevent sinistral portal hypertension [17]. However, the beneficial effect of drainage via these critical veins is greatly affected by individual anatomy [17], and it is often difficult to preserve these critical veins in patients with advanced pancreatic cancer.

Most surgeons currently believe that the splenic venous flow must be reconstructed in cases where the splenic vein is divided [15–20]. Although some venous reconstructions (e.g., anastomoses to the inferior mesenteric vein or direct implantation to the superior mesenteric vein and portal vein) have been already documented [12, 15, 20], these reconstructions have limitations regarding feasibility and long-term patency. Hence, we consider that the splenic vein should be antegradely reconstructed into a thick vein (i.e., the left renal vein), and so we created a distal splenorenal shunt for splenic venous reconstruction since 2014.

Warren was a pioneer in surgery for portal hypertension and variceal bleeding and originally published the distal splenorenal shunt in 1967 as an operative procedure for bleeding esophageal varices [27]. Warren originally established the distal splenorenal shunt for patients with cirrhosis, and operation in cirrhotic patient needs advanced surgical skill in handling the splenic vein with high pressure and spleno-pancreatic disconnection in necessary to maintain selectivity of portal perfusion. Creation of distal splenorenal shunt in noncirrhotic patients is much easier and safer, because these troublesome procedures are unnecessary. We therefore considered the distal splenorenal shunt safe and feasible in patients without liver cirrhosis. Other surgeons employed distal splenorenal shunt for intentional decompression of the splenic vein during pancreatic surgery in cirrhotic patient [36], we have a clear impression that a creation of splenorenal shunt is easier than that in cirrhotic patient. Splenorenal shunt has a safety and feasibility for intentional decompression of the splenic vein. On the other hand, we made distal splenorenal shunt first, in order to avoid even a subtle splenic congestion during surgery. We did not use vessel prosthesis, because of pancreatic juice-related complication after surgery.

To our knowledge, there are only few reported cases of splenic venous reconstruction via a distal splenorenal shunt [12, 37–39]. Moreover, no report has focused on the long-term effects of a distal splenorenal shunt to prevent sinistral portal hypertension, as pancreatic cancer has a very poor prognosis [3–6]. In the present case, we used a distal splenorenal shunt for splenic venous reconstruction during radical surgery for carcinoma of the head of the pancreas with regional invasion, and close long-term follow-up revealed venous anastomotic patency with no recurrence. Currently, tumor biology and risk factor for pancreatic cancer are detected [40, 41]. An exceptional long-term survival of our patient might be probably related to a favorable tumor biology.

In conclusion, this case has shown the importance of early diagnosis of carcinoma of the head of the pancreas, as appropriate and timely surgical management can result in good outcome. This patient responded well and remains alive six years following pancreaticoduodenectomy and preservation of the spleen with the use of a distal splenorenal shunt.

## Data Availability

All data were clearly shown in figures and text.
